# Supplementary material for: MicroPIPE: validating an end-to-end workflow for high-quality complete bacterial genome construction
Source: BMC Genomics. 2021 Jun 25;22:474. doi: 10.1186/s12864-021-07767-z (PMC8235852; doi:10.1186/s12864-021-07767-z)
Supplement: Supplementary file 1 — Additional file 1: Table S1. Read length and quality metrics per isolate. Table S2. assembly results for EC958. Table S3. EC958 assembly results using different Flye parameters, demultiplexing tools and read filtering parameters. Table S4. Polishing tool comparison Table S5. Polishing comparison using other assemblers Table S6. Hybrid assembly comparison to Flye+Racon/medaka+NextPolish Table S7. final assembly comparisons between Guppy versions and methylation-aware basecalling. Table S8. MicroPIPE v0.8 results for 11 ST131. Table S9. SNP types for clade C unpolished/Illumina unpolished assemblies. Table S10. Demultiplexing comparison between qcat and Guppy Table S11. Assembly comparison using different filtering parameters(qcat demultiplexing). Table S12. Assembly comparison using different filtering parameters (guppy demultiplexing). Table S13. Assembly comparison using ONT raw or corrected reads Figure S1. Demultiplexing metrics. Figure S2. Long-read metrics using different demultiplexing tools and read filtering parameters (using EC958 ONT data). Figure S3. comparison of SNPs/indels in ONT assemblies vs. complete EC958 chromosome. Figure S4. Motif enriched in the sequences around the 401 shared SNPs from the branch leading to discrepant ONT assemblies. [file 12864_2021_7767_MOESM1_ESM.docx]

**Supplementary Materials**

**Supplementary Table 1: Read length and quality metrics per isolate**

| **Sample** | **Number**  **of Reads** | **Number**  **of Bases** | **Read Length**  **N50** | **Median**  **Read Length** | **Median**  **Read Quality** |
| --- | --- | --- | --- | --- | --- |
| S24 | 83,023 | 518,350,475 | 11,366 | 3,870 | 11.7 |
| S34 | 130,069 | 998,292,413 | 13,795 | 4,900 | 11.6 |
| S37 | 82,516 | 524,209,315 | 11,692 | 3,938 | 11.6 |
| S39 | 127,098 | 1,021,807,859 | 14,852 | 4,933 | 11.6 |
| S65 | 93,069 | 701,752,024 | 13,675 | 4,742 | 11.7 |
| S96 | 138,299 | 901,024,145 | 11,778 | 4,111 | 11.6 |
| S97 | 149,425 | 1,138,211,010 | 13,728 | 4,820 | 11.7 |
| S112 | 91,655 | 705,206,951 | 14,213 | 4,740 | 11.6 |
| S116 | 111,062 | 900,213,042 | 14,681 | 5,131 | 11.6 |
| S129 | 66,930 | 505,613,353 | 14,242 | 4,470 | 11.6 |
| EC958 | 242,401 | 1,755,197,084 | 13,131 | 4,577 | 11.6 |
| HVM2044 | 117,328 | 778,919,514 | 12,001 | 4,145 | 11.5 |

**Supplementary Table 2: assembly results for EC958**

| Assembler | Runtime (min) | Number of contigs | Size (bp) | Nucleotide Identity (%) | Number of misassemblies and local misassemblies | Genome fraction (%) | Circular? |
| --- | --- | --- | --- | --- | --- | --- | --- |
| Flye v2.5 | 46 | 4 | 5,130,397  136,261  4,243  1,841 | 99.41 | 2  1 | 100 | Yes  Yes  Yes  Yes |
| Canu v1.9 | 168 | 5 | 5,091,050 *  135,078 *  4,078 *  1,814 *  1,898 | 99.60 | 4  4 | 99.873 | Yes  Yes  Yes  Yes (nucmer)  No |
| Unicycler (long-read) v0.4.7 | 83 | 6 | 5,104,112  133,747  111,708  107,098  57,320  4,085 | 99.71 | 13  3 | 99.961 | Yes  Yes  No  No  No  Yes |
| Raven v1.1.5 | 19 | 2 | 5,103,450  135,356 | 99.73 | 0  1 | 99.863 | Yes  Yes |
| Redbean v2.5 | 8.5 | 2 | 5,089,255  135,095 * | 99.59 | 2  2 | 99.831 | Yes (manual)  Yes (manual) |
| Shasta v0.4.0 | 4.5 | 2 | 5,113,702  135,731 | 99.31 | 0  9 | 99.921 | Yes  Yes |

* : Sizes correspond to contigs after manual trimming of overlapping ends

**Supplementary Table 3: EC958 assembly results using different Flye parameters, demultiplexing tools and read filtering parameters:** This table summarised the number of contigs from the Flye assembly and the number of contigs reported as circular using different read filtering parameters, different demultiplexing tools and different Flye parameters. The numbers represent “number of circular contigs” : “total number of contigs assembled”. *Note:* the expected number of contigs for the complete EC958 genome is 4 (1 chromosome, and 3 plasmids). Any assemblies with less than 4 total contigs were found to have plasmids that were not assembled.

| **Filtering** | **Demultiplexing** | **Number of reads** | **Number of circular contigs : Number of contigs** | | |
| --- | --- | --- | --- | --- | --- |
|  |  |  | **Flye (default)** | **Flye --plasmids** | **Flye**  **--plasmids --meta** |
| All reads  (Porechop trimmed) | Deepbinner | 182,607 | 2:2 | 4:4 | 4:4 |
|  | Guppy | 226,464 | 2:2 | 4:4 | 4:4 |
|  | Qcat | 238,000 | 2:2 | 4:4 | 4:4 |
| Japsa | Deepbinner | 153,573 | 2:2 | 4:4 | 4:4 |
| --lenMin 1000 | Guppy | 190,440 | 2:2 | 4:4 | 4:4 |
| --qualMin 10 | Qcat | 200,148 | 2:2 | 4:4 | 4:4 |
| Japsa | Deepbinner | 131,623 | 2:2 | 3:3 | 3:3 |
| --lenMin 2000 | Guppy | 163,137 | 2:2 | 3:3 | 3:3 |
| --qualMin 5 | Qcat | 171,456 | 2:2 | 3:3 | 3:3 |
| Filtlong | Deepbinner | 104,487 | 2:2 | 3:3 | 3:3 |
| --min_length 1000 | Guppy | 129,170 | 2:2 | 3:3 | 3:3 |
| --keep_percent 90 | Qcat | 135,791 | 2:2 | 3:3 | 3:3 |

**Supplementary Table 4: Polishing tool comparison:** Racon/Medaka + NextPolish (green) was selected for MicroPIPE

|  | Read set | Run time (min) | DNAdiff | | | Pomoxis | | | QUAST | |
| --- | --- | --- | --- | --- | --- | --- | --- | --- | --- | --- |
|  |  |  | Nucleotide Identity (%) | SNPs | Indels | Quality  score | Identity  quality  score | Indel quality score | Mismatches  per 100 kb | Indels  per 100 kb |
| Racon/Medaka | Long | 92 | 99.79 | 7199 | 3536 | 26.85 | 28.65 | 34.56 | 136.57 | 67.66 |
| Racon/Medaka + Pilon | Long and short | 113 | 99.99 | 45 | 61 | 46.34 | 50.6 | 51.86 | 0.86 | 1.18 |
| Racon/Medaka + NextPolish | Long and short | 97 | 99.99 | 23 | 45 | 48.10 | 53.52 | 53.14 | 0.44 | 0.88 |
| Nanopolish | Long | 261 | 99.82 | 1512 | 7807 | 27.43 | 35.46 | 32.74 | 28.35 | 148.83 |
| Nanopolish + Pilon | Long and short | 277 | 99.99 | 69 | 109 | 44.35 | 49.42 | 49.55 | 1.07 | 2.1 |
| Nanopolish + NextPolish | Long and short | 266 | 99.99 | 51 | 67 | 45.93 | 51.01 | 50.82 | 0.72 | 1.3 |
| NextPolish | Long | 2.5 | 99.67 | 8108 | 8367 | 24.81 | 28.13 | 31.61 | 153.43 | 159.94 |
| NextPolish | Short | 5 | 99.99 | 75 | 127 | 43.26 | 49.14 | 48.2 | 1.22 | 2.42 |
| NextPolish | Long and short | 6.5 | 99.99 | 44 | 52 | 46.92 | 50.9 | 52.85 | 0.8 | 1.03 |
| Pilon | Short | 19 | 99.99 | 100 | 222 | 41.42 | 47.69 | 46.75 | 1.7 | 4.23 |

**Supplementary Table 5: Polishing comparison using other assemblers (Long read assembly followed by polishing with Racon/Medaka+NextPolish [blue] and hybrid assembly [yellow]):** top three in each quality category highlighted green

| Assembler | Assembly strategy | DNAdiff | | | Pomoxis | | | QUAST | | | | |  |
| --- | --- | --- | --- | --- | --- | --- | --- | --- | --- | --- | --- | --- | --- |
|  |  | Nucleotide Identity (%) | SNPs | Indels | Quality score | Identity quality score | Indel quality score | Mismatches per 100 kb | Indels per 100 kb | Indels length | Genome fraction (%) | Duplication Ratio |  |
| Flye v2.5 | Long read assembly + polishing | 99.99 | 23 | 45 | 48.10 | 53.52 | 53.14 | 0.44 | 0.88 | 58 | 100 | 1 |  |
| Canu v1.9 | Long read assembly + polishing | 99.99 | 51 | 41 | 45.23 | 50.35 | 50.36 | 2.48 | 2.5 | 185 | 99.952 | 1.021 |  |
| Unicycler v0.4.7 | Long read assembly + polishing | 99.99 | 78 | 65 | 45.54 | 48.58 | 52.01 | 16.26 | 18.47 | 1469 | 99.949 | 1.049 |  |
| Raven v1.1.5 | Long read assembly + polishing | 99.99 | 28 | 49 | 48.25 | 52.7 | 53.88 | 0.53 | 0.93 | 55 | 99.922 | 1 |  |
| Redbean v2.5 | Long read assembly + polishing | 99.99 | 28 | 56 | 45.74 | 50.64 | 51.93 | 0.92 | 1.01 | 184 | 99.831 | 1 |  |
| Shasta v0.4.0 | Long read assembly + polishing | 99.99 | 91 | 38 | 46.88 | 49.89 | 53.68 | 1.66 | 0.82 | 79 | 99.922 | 1 |  |
| Unicycler v0.4.7 | Hybrid assembly | 99.99 | 165 | 34 | 41.38 | 43.06 | 50.37 | 3.11 | 0.61 | 159 | 100 | 1 |  |
|  | Hybrid assembly + polishing | 99.99 | 38 | 45 | 44.10 | 45.68 | 52.54 | 0.88 | 0.91 | 63 | 100 | 1 |  |
| MaSuRCA v3.3.5 | Hybrid assembly | 99.99 | 67 | 15 | 39.15 | 49.00 | 42.67 | 1.35 | 0.31 | 32 | 99.922 | 1.022 |  |
|  | Hybrid assembly + polishing | 99.98 | 63 | 49 | 40.77 | 43.01 | 47.95 | 4.94 | 5.66 | 412 | 99.917 | 1.021 |  |
| SPAdes v3.13.1 | Hybrid assembly | 99.98 | 1073 | 87 | 35.31 | 37.03 | 43.66 | 20.92 | 1.83 | 562 | 99.960 | 1 |  |
|  | Hybrid assembly + polishing | 99.99 | 43 | 37 | 48.10 | 51.06 | 54.67 | 0.78 | 0.74 | 95 | 99.962 | 1 |  |

**Supplementary Table 6: Hybrid assembly comparison to Flye+Racon/medaka+NextPolish:** Flye + polishing (green) was selected for MicroPIPE

|  | Runtime (min) | Number of contigs | Size of contigs (bp) | Circularised? | DNAdiff (nucleotide identity) | Number of misassemblies | Genome fraction (%) |
| --- | --- | --- | --- | --- | --- | --- | --- |
| Flye v2.5 only assembly | 46 | 4 | 5,130,406  136,260  4,245  1,841 | Yes  Yes  Yes  Yes | 99.41 | 2 | 100 |
| Flye v2.5 + polishing | 143 | 4 | 5,109,793  135,596  4,208  1,823 | Yes  Yes  Yes  Yes | 99.99 | 2 | 100 |
| Unicycler v0.4.7 (hybrid) | 360 | 4 | 5,109,706  135,600  4,088  1,822 | Yes  Yes  Yes  Yes | 99.99 | 4 | 100 |
| MaSuRCA v3.3.5 | 55 | 2 | 5,109,995 *  135,599 * | Yes (nucmer)  Yes (nucmer) | 99.99 | 2 | 99.922 |
| SPAdes v3.13.1 | 70 | 5 | 2,937,224  2,171,161  134,802  4,088 *  1,822 * | No  No  No  Yes (manual)  Yes (manual) | 99.98 | 0 | 99.960 |

* Sizes correspond to contigs after manual trimming of overlapping ends

**Supplementary Table 7: final assembly comparisons between Guppy versions and methylation-aware basecalling**

| Guppy version | Guppy model | Data used for polishing | Nb SNPs | Nb Indels | Nb Total |
| --- | --- | --- | --- | --- | --- |
| v3.4.3 | hac | ONT | 7,199 | 3,536 | 10,735 |
|  |  | ONT + Illumina | 23 | 45 | 68 |
|  | modbases_hac | ONT | 160 | 1,997 | 2,157 |
|  |  | ONT + Illumina | 3 | 31 | 34 |
| v3.6.1 | hac | ONT | 28 | 438 | 466 |
|  |  | ONT + Illumina | 4 | 25 | 29 |
|  | modbases_hac | ONT | 117 | 1,663 | 1,780 |
|  |  | ONT + Illumina | 5 | 27 | 32 |

**Supplementary Table 8: MicroPIPE v0.8 results for 11 ST131**

| Strain | Chromosome/plasmid | Size (bps) | Circularised? |
| --- | --- | --- | --- |
| S24EC | Chromosome  Plasmid A | 5,061,955  114,236 | Yes  Yes |
| S34EC | Chromosome  Plasmid A  Plasmid B | 5,034,986  152,718  107,794 | Yes  Yes  Yes |
| S37EC | Chromosome  Plasmid A  Plasmid B | 4,965,673  157,040  60,747 | Yes  Yes  Yes |
| S39EC | Chromosome  Plasmid A  Plasmid B  Plasmid C  Plasmid D  Plasmid E  Plasmid F | 5,038,121  143,728  94,575  66,312  61,832  2,018  1,788 | Yes  Yes  Yes  Yes  Yes  Yes  Yes |
| S65EC | Chromosome  Plasmid A | 5,187,864  146,795 | Yes  Yes |
| S96EC | Chromosome  Plasmid A  Plasmid B  Plasmid C  Plasmid D | 5,052,350  163,696  115,542  14,053  4,073 | Yes  Yes  Yes  Yes  Yes |
| S97EC | Chromosome  Plasmid A  Plasmid B  Plasmid C  Plasmid D | 5,162,367  165,456  96,393  4,059  3,185 | Yes  Yes  Yes  Yes  Yes |
| S112EC | Chromosome  Plasmid A  Plasmid B  Plasmid C  Plasmid D | 5,003,915  160,386  34,185  5,208  4,141 | Yes  Yes  Yes  Yes  Yes |
| S116EC | Chromosome  Plasmid A  Plasmid B  Plasmid C  Plasmid D | 4,972,973  66,522  5,183  4,122  4,117 | Yes  Yes  Yes  Yes  Yes |
| S129EC | Chromosome  Plasmid A  Plasmid B  Plasmid C  Plasmid D  Plasmid E  Plasmid F  Plasmid G | 5,177,746  163,021  93,601  33,138  4,071  2,399  2,108  1,555 | Yes  Yes  Yes  Yes  Yes  Yes  Yes  Yes |
| HVM2044 | Chromosome  Plasmid A  Plasmid B  Plasmid C  Plasmid D | 4,986,664  142,349  115,439  18,084  6,772 | Yes*  No  Yes  Yes  No |

* modifying filtering parameters resulted in 5 circular contigs (Filtlong –min_length 1000 –keep_percent 90)

**Supplementary Table 9: SNP types for clade C unpolished/Illumina unpolished assemblies**

| **SNP type** | **Allele change (ref → alt)** | **Count** |
| --- | --- | --- |
| **Transition**  399 | A → G | 187 |
|  | G → A | 3 |
|  | T → C | 203 |
|  | C → T | 6 |
| **Transversion**  2 | A → T | 0 |
|  | T → A | 0 |
|  | T → G | 0 |
|  | G → T | 0 |
|  | G → C | 1 |
|  | C → G | 1 |
|  | C → A | 0 |
|  | A → C | 0 |

**Supplementary Table 10: Demultiplexing comparison between qcat and Guppy: run-times, EC958 read and assembly accuracy**

| **Basecalling comparison** | **Guppy3.4.3_hac** | | **Guppy3.6.1_hac** | |
| --- | --- | --- | --- | --- |
| **Demultiplexing tool** | **qcat** | **guppy** | **qcat** | **guppy** |
| Demultiplexing run time (h) | 0.58 | 0.68 | 0.56 | 0.47 |
| Average read percent identity | 91.0 | 91.2 | 93.7 | 93.8 |
| Mean read quality | 11.4 | 11.5 | 13.3 | 13.4 |
| Read length N50 | 13,092 | 13,087 | 13,021 | 13,013 |
| Number of binned reads | 240,766 | 229,100 | 244,830 | 240,124 |
| **Final assembly comparison** |  | | | |
| Assembly nucleotide identity (%) | 99.99 | 99.99 | 99.99 | 99.99 |
| Number of SNP (DNAdiff) | 23 | 28 | 4 | 4 |
| Number of GSNP (DNAdiff) | 3 | 6 | 1 | 1 |
| Number of indels (DNAdiff) | 45 | 35 | 25 | 25 |
| Assembly quality score (Pomoxis) | 48.10 | 48.76 | 52.27 | 52.41 |
| Mismatches per 100 kb (QUAST) | 0.44 | 0.53 | 0.08 | 0.08 |
| Indels per 100 kb (QUAST) | 0.88 | 0.69 | 0.50 | 0.48 |
| Contig Size (bp) | 5,109,793  135,596  4,208  1,823 | 5,109,791  135,600  4,103  1,855 | 5,109,781  135,600  4,103  1,820 | 5,109,776  135,600  4,100  1,809 |

**Supplementary Table 11: Assembly comparison using different filtering parameters: EC958 read and assembly accuracy (qcat demultiplexing)**

| **Basecalling** | **Guppy3.4.3_hac** | | | | **Guppy3.6.1_hac** | | | |
| --- | --- | --- | --- | --- | --- | --- | --- | --- |
| **Filtering parameter** | All reads  (Porechop trimmed) | Japsa  --lenMin 1000  --qualMin 10 | Japsa  --lenMin 2000  --qualMin 5 | Filtlong  --min_length 1000  --keep_percent 90 | All reads  (Porechop trimmed) | Japsa  --lenMin 1000  --qualMin 10 | Japsa  --lenMin 2000  --qualMin 5 | Filtlong  --min_length 1000  --keep_percent 90 |
| Number of reads | 239,123 | 202,759 | 175,104 | 138,826 | 242,794 | 205,223 | 176,916 | 139,921 |
| Average read percent identity | 91.0 | 90.8 | 90.8 | 91.0 | 93.7 | 93.6 | 93.5 | 93.7 |
| Mean read quality | 11.5 | 11.5 | 11.5 | 11.7 | 13.6 | 13.6 | 13.5 | 13.7 |
| Read length N50 | 13,106 | 13,249 | 13,557 | 14,393 | 13,042 | 13,181 | 13,489 | 14,321 |
| **Final assembly comparison** |  |  | | | | | | |
| Assembly nucleotide identity (%) | 99.99 | 99.99 | 99.99 | 99.99 | 99.99 | 99.99 | 99.99 | 99.99 |
| Number of SNP (DNAdiff) | 35 | 23 | 26 | 33 | 2 | 4 | 4 | 2 |
| Number of GSNP (DNAdiff) | 6 | 3 | 3 | 4 | 1 | 1 | 1 | 1 |
| Number of indels (DNAdiff) | 41 | 45 | 46 | 44 | 26 | 25 | 27 | 27 |
| Assembly quality score (Pomoxis) | 48.32 | 48.10 | 48.98 | 48.27 | 52.67 | 52.27 | 53.52 | 51.87 |
| Mismatches per 100 kb (QUAST) | 0.67 | 0.44 | 0.50 | 0.63 | 0.04 | 0.08 | 0.08 | 0.04 |
| Indels per 100 kb (QUAST) | 0.78 | 0.88 | 0.90 | 0.86 | 0.51 | 0.50 | 0.53 | 0.53 |
| Contig Size (bp) | 5,109,762  135,600  4,284  1,610 | 5,109,793  135,596  4,208  1,823 | 5,109,797  135,600  4,180 | 5,109,762  135,600  4,093 | 5,109,782  135,600  4,100  1,810 | 5,109,781  135,600  4,103  1,820 | 5,109,781  135,600  4,116 | 5,109,783  135,598  4,098 |

**Supplementary Table 12: Assembly comparison using different filtering parameters: EC958 read and assembly accuracy (guppy demultiplexing)**

| **Basecalling** | **Guppy3.4.3_hac** | | | | **Guppy3.6.1_hac** | | | |
| --- | --- | --- | --- | --- | --- | --- | --- | --- |
| **Filtering parameter** | All reads  (Porechop trimmed) | Japsa  --lenMin 1000  --qualMin 10 | Japsa  --lenMin 2000  --qualMin 5 | Filtlong  --min_length 1000  --keep_percent 90 | All reads  (Porechop trimmed) | Japsa  --lenMin 1000  --qualMin 10 | Japsa  --lenMin 2000  --qualMin 5 | Filtlong  --min_length 1000  --keep_percent 90 |
| Number of reads | 227,542 | 192,927 | 166,613 | 132,076 | 238,139 | 201,130 | 173,392 | 137,395 |
| Average read percent identity | 91.1 | 91.0 | 91.0 | 91.2 | 93.8 | 93.7 | 93.6 | 93.8 |
| Mean read quality | 11.6 | 11.6 | 11.6 | 11.7 | 13.7 | 13.6 | 13.6 | 13.8 |
| Read length N50 | 13,102 | 13,240 | 13,547 | 14,387 | 13,031 | 13,173 | 13,480 | 14,301 |
| **Final assembly comparison** |  |  | | | | | | |
| Assembly nucleotide identity (%) | 99.99 | 99.99 | 99.99 | 99.99 | 99.99 | 99.99 | 99.99 | 99.99 |
| Number of SNP (DNAdiff) | 24 | 28 | 29 | 43 | 4 | 4 | 4 | 2 |
| Number of GSNP (DNAdiff) | 4 | 6 | 9 | 7 | 1 | 1 | 1 | 1 |
| Number of indels (DNAdiff) | 37 | 35 | 38 | 46 | 24 | 25 | 22 | 26 |
| Assembly quality score (Pomoxis) | 45.88 | 48.76 | 48.93 | 47.56 | 52.70 | 52.41 | 52.83 | 52.54 |
| Mismatches per 100 kb (QUAST) | 0.46 | 0.53 | 0.55 | 0.82 | 0.08 | 0.08 | 0.08 | 0.04 |
| Indels per 100 kb (QUAST) | 0.72 | 0.69 | 0.74 | 0.88 | 0.48 | 0.48 | 0.46 | 0.51 |
| Contig Size (bp) | 5,109,787  135,601  4,110  1,833 | 5,109,791  135,600  4,103  1,855 | 5,109,769  135,600  4,108 | 5,109,795  135,600  4,190 | 5,109,777  135,600  4,103  1,825 | 5,109,776  135,600  4,100  1,809 | 5,109,779  135,600  4,092 | 5,109,782  135,598  4,093 |

**Supplementary Table 13: Assembly comparison using ONT raw or corrected reads: run-times, EC958 read and assembly accuracy**

Read correction was performed using Canu. Flye was run using the --nano-raw parameter (raw reads) or the --nano-corr parameter (corrected reads).

| **Basecalling comparison** | **Guppy3.4.3_hac** | | **Guppy3.6.1_hac** | | **Guppy4.4.1_hac** | |
| --- | --- | --- | --- | --- | --- | --- |
| **ONT dataset** | **Raw reads** | **Corrected reads** | **Raw reads** | **Corrected reads** | **Raw reads** | **Corrected reads** |
| Read correction run time (min) | 0 | 236 | 0 | 206 | 0 | 246 |
| Assembly run time (min) | 64 | 15 | 68 | 16 | 72 | 17 |
| Polishing run time (min) | 43 | 35 | 30 | 33 | 38 | 33 |
| Total run time (min) | 107 | 286 | 98 | 255 | 110 | 296 |
| **Final assembly comparison** |  | |  | |  | |
| Assembly nucleotide identity (%) | 99.99 | 99.99 | 99.99 | 99.99 | 99.99 | 99.99 |
| Number of SNP (DNAdiff) | 23 | 71 | 4 | 2 | 4 | 4 |
| Number of GSNP (DNAdiff) | 3 | 6 | 1 | 1 | 1 | 1 |
| Number of indels (DNAdiff) | 45 | 45 | 25 | 20 | 23 | 26 |
| Assembly quality score (Pomoxis) | 48.10 | 46.28 | 52.27 | 53.75 | 53.02 | 52.38 |
| Mismatches per 100 kb (QUAST) | 0.44 | 1.35 | 0.08 | 0.04 | 0.08 | 0.08 |
| Indels per 100 kb (QUAST) | 0.88 | 0.88 | 0.50 | 0.40 | 0.46 | 0.51 |
| Contig Size (bp) | 5,109,793  135,596  4,208  1,823 | 5,109,782  135,601  4,090  1,823 | 5,109,781  135,600  4,103  1,820 | 5,109,776  135,592  4,100  1,824 | 5,109,775  135,598  4,085  1,816 | 5,109,781  135,600  4,091  1,819 |

**Supplementary Figures:**

**
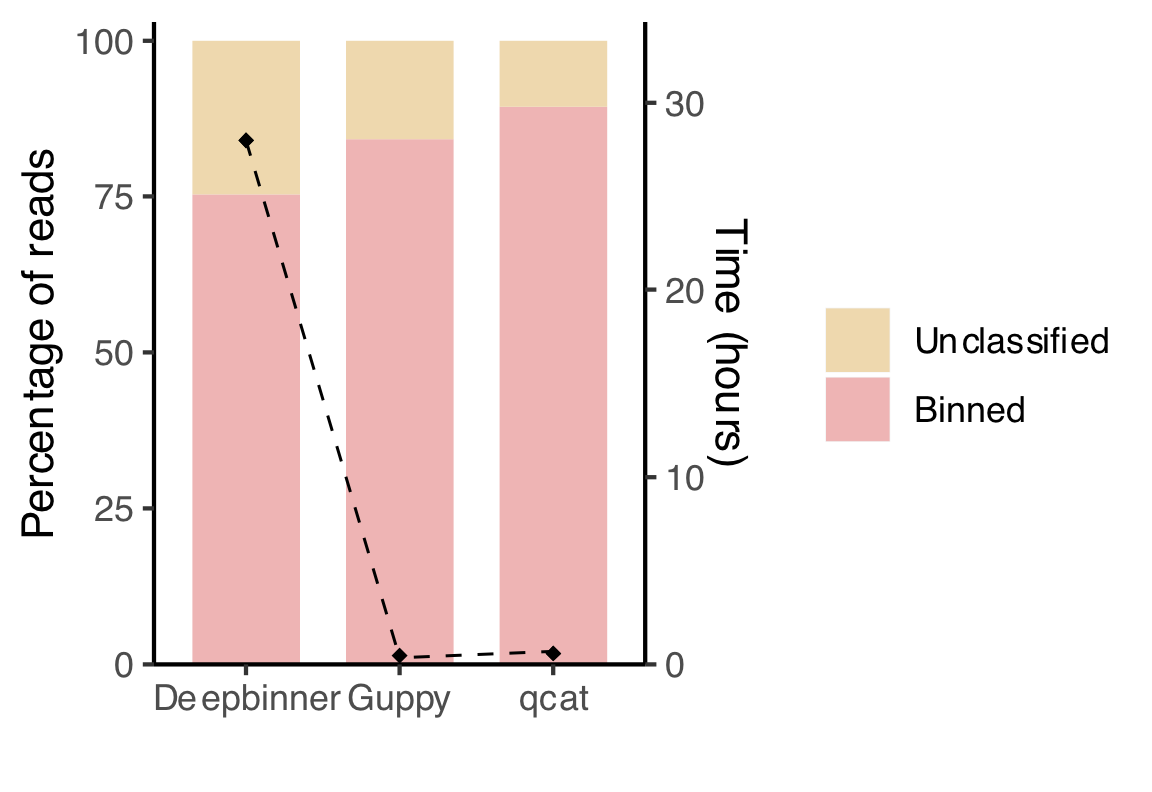
**

**Supplementary Figure 1: Demultiplexing metrics**

**Supplementary Figure 2: Long-read metrics using different demultiplexing tools and read filtering parameters (using EC958 ONT data)**

**
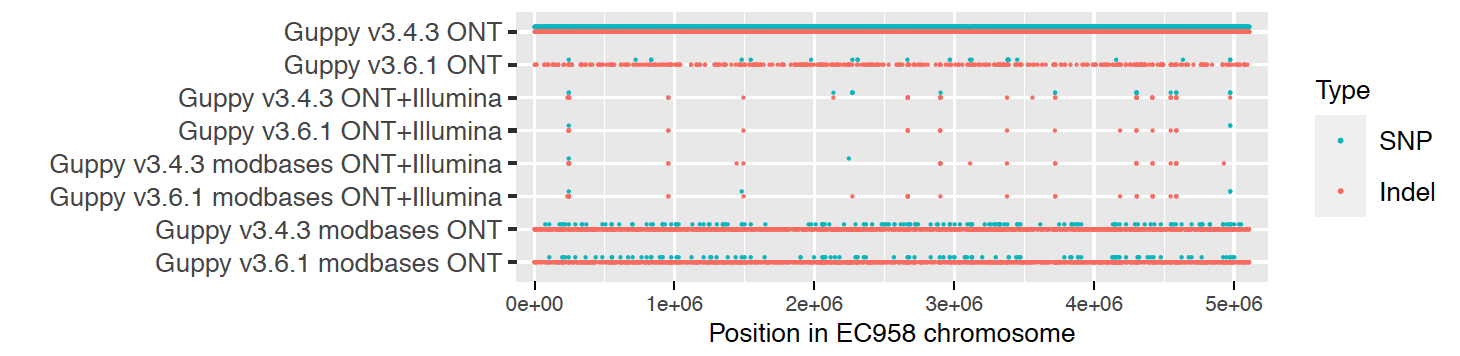
**

**Supplementary Figure 3: comparison of SNPs/indels in ONT assemblies vs. complete EC958 chromosome**


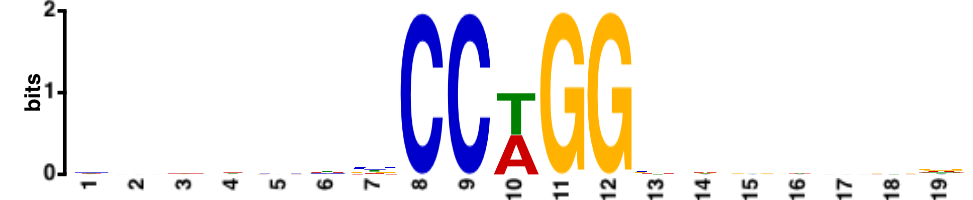


**Supplementary Figure 4: Motif enriched in the sequences around the 401 shared SNPs from the branch leading to discrepant ONT assemblies as indicated by the star in Figure 5A (main text).**
